# Bowel obstruction associated with a feeding jejunostomy and its association to weight loss after thoracoscopic esophagectomy

**DOI:** 10.1186/s12876-019-1029-6

**Published:** 2019-06-25

**Authors:** Hiroyuki Kitagawa, Tsutomu Namikawa, Jun Iwabu, Sunao Uemura, Masaya Munekage, Keiichiro Yokota, Michiya Kobayashi, Kazuhiro Hanazaki

**Affiliations:** 1 Department of Surgery, Kochi Medical School, Kohasu-Okocho, Nankoku, Kochi 783-8505 Japan; 2 Department of Human Health and Medical Sciences, Kochi Medical School, Kohasu-Okocho, Nankoku, Kochi 783-8505 Japan

**Keywords:** Feeding jejunostomy, Bowel obstruction associated with a feeding jejunostomy, Body weight loss, Thoracoscopic esophagectomy, Esophageal cancer

## Abstract

**Background:**

Our aim was to clarify the incidence of bowel obstruction associated with a feeding jejunostomy (BOFJ) after thoracoscopic esophagectomy and its association to characteristics and postoperative change in body weight.

**Methods:**

We reviewed 100 consecutive patients who underwent thoracoscopic esophagectomy with gastric tube reconstruction and placement of a jejunostomy feeding catheter for esophageal cancer. The incidence of BOFJ was evaluated and the change in body weight after surgery was compared between patients *with* and *without* BOFJ.

**Results:**

BOFJ developed in 17 patients. Compared to patients *without* BOFJ, those *with* BOFJ had a higher preoperative body mass index (23.3 kg/m^2^ versus 20.9 kg/m^2^, *P* = 0.022), and greater postoperative body weight loss rate: 3 month, decrease to 84.2% of initial body weight versus 89.3% (*P* = 0.002). Patients *with* BOFJ had shorter distance between the jejunostomy and midline (40 mm versus 48 mm, *P* = 0.011) compared to patients *without* BOFJ. On multivariate analysis, higher preoperative body mass index (odds ratio (OR) = 9.248; 95% confidence interval (CI) = 1.344–63.609; *p* = 0.024), higher postoperative weight loss at 3 months (OR = 8.490; 95% CI = 1.765–40.837, *p* = 0.008), and shorter distance between the jejunostomy and midline (OR = 8.160; 95% CI = 1.675–39.747, *p* = 0.009) were independently associated with BOFJ.

**Conclusion:**

Patients of BOFJ had greater preoperative body mass, shorter distance between jejunostomy and midline, and greater postoperative weight loss.

## Background

Esophagectomy with radical lymphadenectomy is the main treatment for esophageal cancer. However, esophagectomy is associated with a high incidence of postoperative complications [1], even when a less invasive thoracoscopic procedure is used [2, 3]. In addition, reconstruction of the gastric tube, which is commonly required with esophagectomy, is associated with a high incidence of anastomotic leakage [1]. From a clinical perspective, postoperative weight loss is common after esophagectomy, even in the absence of any complications [4], with severe weight loss being associated with a poor prognosis [5].

Early enteral nutrition after esophagectomy is recommended, with insertion of a feeding catheter during the esophagectomy being useful for an appropriate nutritional strategy after surgery [6, 7]. As per previously published methods, we routinely create a feeding jejunostomy during esophagectomy in our institution, using a catheter, and initiate enteral nutrition on postoperative day 1 [4]. As well, patients continue with enteral nutritional supplementation after discharge until their dietary intake is sufficient. Despite this aggressive nutritional strategy, more than half of patients experience a > 10% weight loss during the first 6 months after surgery [4]. Moreover, the feeding catheter can sometimes cause bowel obstruction, requiring emergent surgery for treatment.

Therefore, the aim of our study was to clarify the incidence of bowel obstruction associated with a feeding jejunostomy (BOFJ) after thoracoscopic esophagectomy (TSE) and to evaluate the association between BOFJ and the patients’ characteristics or postoperative course of change in body weight (BW).

## Methods

This was a retrospective observational study of 100 consecutive patients who underwent TSE for esophageal cancer, followed by gastric tube reconstruction, with placement of a jejunostomy feeding catheter, at our institution, between July 2009 and May 2017. Patients treated using a lower esophagectomy, via an abdominal approach, were excluded. Preoperatively, all patients underwent a comprehensive examination, including endoscopy, computed tomography (CT), barium swallow radiography, and biochemical blood tests. Neo-adjuvant chemotherapy, using cisplatin and fluorouracil, *with* or *without* docetaxel, was administered to patients diagnosed with clinical stage II, III and IV cancer, as per the result of Japanese clinical study [8].

Thoracoscopic McKeown esophagectomy with mediastinal dissection was performed in prone position. After thoracoscopic esophagectomy, patients were placed in supine position, gastric mobilization with abdominal dissection and gastric tube reconstruction was performed.

Informed consent was omitted and information of this study was disclosed in the form of opt-out on our hospital website.

### Insertion of a feeding catheter in the jejunum

A 30 cm, 9 Fr, feeding catheter (Kangaroo Jejunostomy Catheter, Covidien Japan, Tokyo, Japan) was inserted via a 7 cm middle incision after laparoscopic gastric mobilization and abdominal lymph nodes dissection into the jejunum, 20 cm distal from Treitz ligament. The catheter was secured using the Witzel procedure, with purse string sutures and three additional sutures using absorbable thread over the catheter. In addition, four fixed peritoneum-jejunum sutures using non-absorbable silk thread were placed around the puncture site in the jejunum.

### Postoperative management

Patients were transferred to the surgical intensive care unit, with mechanical ventilation provided for the first night. On the morning of postoperative day 1, patients were weaned off the ventilator and the rehabilitation program initiated. Enteral nutrition, using a liquid diet via the feeding jejunostomy catheter, was also initiated on postoperative day 1, with a caloric intake of 30 kcal/h. The thoracic drain tube was removed on postoperative day 5–7, and oral intake was initiated on postoperative day 7, in the absence of any evidence of anastomotic leakage [9]. Patients were discharged when they were comfortable with oral intake; follow-up visits were scheduled at the hospital at 1, 3, 6, 9, and 12 months after surgery, with subsequent follow-up every 3 to 6 months for an additional year. Patients were advised to maintain an enteral nutrition of 200 to 600 kcal/day, via the feeding catheter, when their daily oral intake was insufficient. The feeding catheter was removed when dietary intake was sufficient to meet nutritional needs.

### Diagnosis of BOFJ

**Fig. 1:**
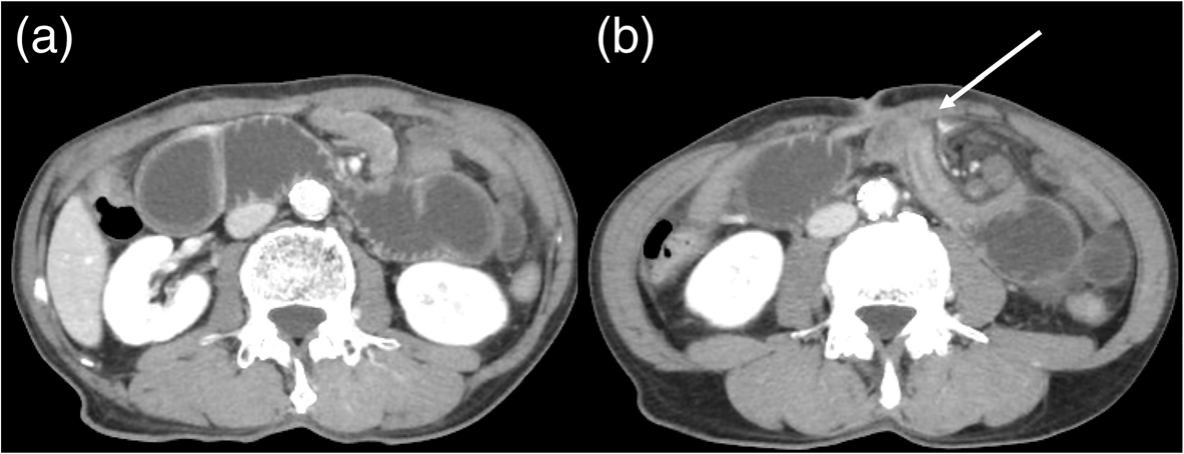
**a.** Computed tomography showing a dilation of the duodenum in a 62-years-old man who had been complaining of acute upper abdominal pain for 18 months after esophagectomy. **b.** An obstruction of the jejunum, at the site of the feeding jejunostomy, was identified (whirl sign; arrow), with twisting of the mesenteric vessels

When the patients complained of acute epigastralgia with a whirl sign visible on CT (Fig. 1a, b) at the site of feeding jejunostomy, we diagnosed as BOFJ. When the whirl sign was not detected on CT, the patients were treated conservatively.

### Outcome parameters

Patients’ characteristics, surgical outcomes and postoperative clinical outcomes were included in the analysis. Patients’ characteristics included: age; sex; cancer histology; clinical cancer stage, according to the 7th edition of the TNM classification [10]; preoperative BW; preoperative body mass index (BMI); and the use of neo-adjuvant chemotherapy. Surgical outcomes included: the use of laparoscopy; the reconstruction method (circular anastomosis or hand-sewn); total operative time (calculated from the time of skin incision to the time of postoperative radiography examination); and total blood loss volume. Postoperative clinical outcomes included: complications, such as pneumonia, anastomotic leakage, recurrent nerve palsy, and surgical site infection; length of hospital stay; and change in body weight, measured at 1, 3, 6, and 12 months after the surgery. In patient’s stature, we calculated the length of the abdominal axis (from xiphoid process to top of pubis), distance between the site of jejunostomy and midline, navel line, and xiphoid process line on CT scan.

### Statistical analysis

For analysis, patients were classified into two groups, the BOFJ group, formed of patients requiring surgery for the treatment of BOFJ after the primary surgery, and the Non-BOFJ group. Patient characteristics, surgical and clinical outcomes and the change in BW after surgery were compared between the two groups. We also analyzed a relationship between the BOFJ and the patient’s stature. Continuous variables are reported as a median and the associated range. The Mann-Whitney U test was used to evaluate differences in continuous variables between the two groups, with Pearson’s chi-squared test used for categorical variables. Kaplan-Meier estimates of accumulated occurrence rate were calculated. Logistic regression analysis was used to identify factors associated with BOFJ. Receiver operating characteristic curve analysis was used to determine the optimal cut-off values for multivariate analysis of patients with BOFJ. All analyses were performed using JMP 13 (SAS Institute Inc., Cary, NC, USA), with a *P*-value < 0.05 considered significant.

## Results

Patient characteristics are reported in Table 1. The median length of postoperative hospital stay was 17.5 days. The median preoperative BW was 56.1 kg, with a postoperative BW at 1, 3, 6, and 12 months of 52.2, 50.0, 49.5, and 51.5 kg, respectively. The median duration between esophagectomy to removal of the feeding catheter was 62 days. The median observation time in this study was 49 months (range; 6–126 months).

**Table 1:** Characteristics of the patients who underwent the thoracoscopic esophagectomy for esophageal cancer

Sex, Male, n (%)	81 (81.0)
Age, years, median (range)	71 (43–85)
Histology, Squamous cell carcinoma, n (%)	85 (85.0)
Stage I / II / III / IV, n	25 / 25 / 37 / 13
Neoadjuvant chemotherapy, n (%)	74 (74.0)
Preoperative body weight, median (range), (kg)	56.1 (40.0–78.0)
Preoperative body mass index, median (range), (kg / m^2^)	21.2 (15.1–30.0)
Laparoscopic procedure, n (%)	87 (87.0)
Anastomosis, circular stapler / hand sewn, n	91 / 9
Operative time, median (range), (min)	612 (456–859)
Blood loss, median (range), (mL)	170 (40–1600)
Complications, n (%)	
Pneumonia	12 (12.0)
Anastomotic leakage	12 (12.0)
Recurrent nerve palsy	28 (28.0)
Surgical site infection	21 (21.0)
Hospital stay, median (range), (days)	17.5 (10–201)
Residual cancer, n (%)	11 (11.0)
Adjuvant therapy, n (%)	
Chemotherapy	45 (45.0)
Chemo-radiotherapy	1 (1.0)
Median postoperative body weight at 1 / 3 / 6 / 12 months after the surgery (kg)	52.2 / 50.0 / 49.5 / 51.5
Median postoperative weight rate at 1 / 3 / 6 / 12 months after the surgery (%)	92.9 / 88.0 / 85.2 / 87.4
Duration until feeding catheter removal, median (range), (days)	62 (6–316)
Surgery for BOFJ, n (%)	17 (17.0)
Duration from esophagectomy to surgery for BOFJ, median (range), (days)	226 (6–1941)
Patient’s stature	
Length of the abdominal axis, median (range), (mm)	330 (265–380)
Distance between the site of jejunostomy and midline, median (range), (mm)	40 (20–70)
Distance between the site of jejunostomy and navel line, median (range), (mm)	30 (0–150)
Distance between the site of jejunostomy and xiphoid process line, median (range), (mm)	110 (50–180)

*BOFJ*; bowel obstruction associated with a feeding jejunostomy

Pathological residual cancer was revealed in 11 patients. Adjuvant chemotherapy was performed for 9 patients, and adjuvant chemo-radiotherapy was performed for 1 patient.

Thirty-six out of 89 non-residual cancer patients were performed the adjuvant chemotherapy. There were no differences of the postoperative body weight change between the patients without and with adjuvant therapy: 1 month, decrease to 93.9% of initial body weight versus 91.8% (*P* = 0.171); 3 months, 89.6% versus 87.6% (*P* = 0.237); 6 months, 87.3% versus 85.0% (*P* = 0.250); and 12 months, 87.6% versus 86.8% (*P* = 0.505).

Cancer recurrence occurred in 26 patients (mediastinal local recurrence, 8; supra-clavicular lymph nodes, 2; intrathoracic dissemination, 2; hematological distant metastasis, 12; distant lymph nodes metastasis, 2). There were no differences in the postoperative body weight change between the patients without and with recurrence: 1 month, decrease to 92.2% of initial body weight versus 93.9% (*P* = 0.748); 3 months, 89.0% versus 87.8% (*P* = 0.910); 6 months, 85.3% versus 85.2% (*P* = 0.573); and 12 months, 87.6% versus 87.0% (*P* = 0.435).

**Fig. 2:**
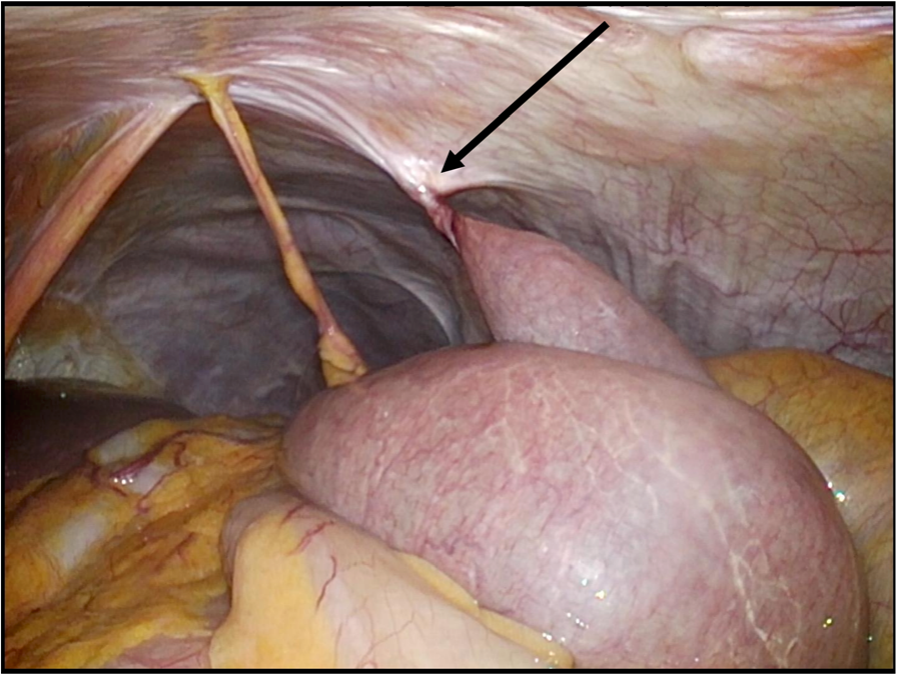
A torsion of the small intestine at the site of feeding jejunostomy was observed by laparoscopy, with the congestion of the jejunum, due to strangulation, improved with relief of the torsion

Postoperative BOFJ developed in 17 of the 100 patients (17%). Nine patients were observed conservatively because they didn’t have any abdominal symptoms although their follow-up CT scan showed the whirl sign. Emergent surgery was required in 9 of these 17 patients for the treatment of acute abdominal pain, with a whirl sign visible on CT (Fig. 1a, b). The other 8 patients required elective surgery for repeated upper abdominal pain, again with a whirl sign visible on CT. Two of 8 patients couldn’t be revealed bowel torsion but adhesion during surgery. The median deration between the esophagectomy and the surgery for BOFJ was 8.4 months (range; 0.2–64.7 months). All 17 patients were treated using adhesiolysis at the jejunostomy site (Fig. 2), with none of the patients requiring a resection of the jejunum. In these 17 patients, 5 had history of abdominal surgery (appendectomy; 2, colorectomy; 2, extended cholecystectomy; 1). Two patients were performed concurrent surgery for hiatus hernia, one had concurrent appendectomy for appendicitis. There was no patient of leakage associated with feeding catheter or accidental removal. However, one patient had skin infection around the catheter. We administrated antibiotics and removed the catheter. During enteral feeding, luminal obstruction of the catheter due to kinking occurred in one patient, then we removed the catheter.

**Fig. 3:**
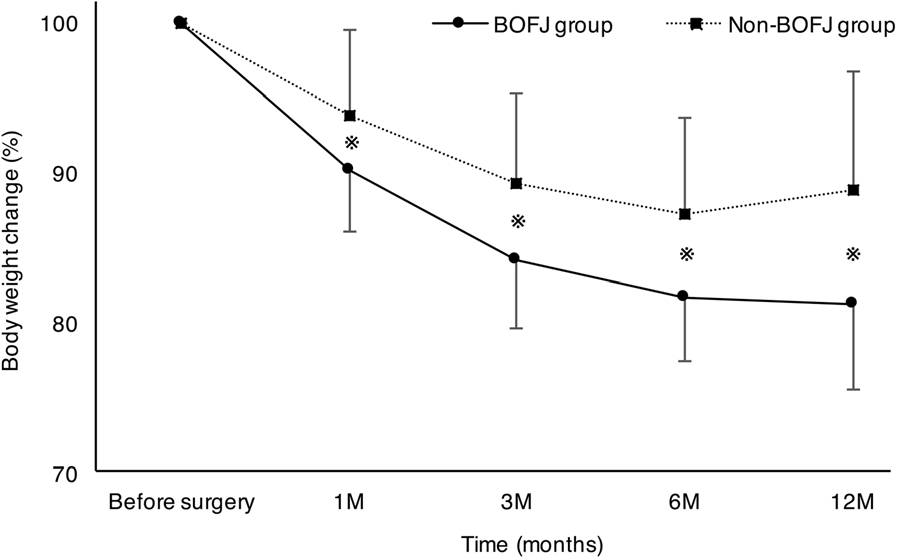
Postoperative change in body weight, expressed as a percentage (%) of the preoperative weight, between the BOFJ group and the Non-BOFJ group, with the asterisk indicating a significant difference between the two groups. In the BOFJ group, weight continued to up to 12 months after surgery. By comparison, in the Non-BOFJ group, weight decreased to 6 months after surgery, with a subsequent increase in weight from 6 to 12 months after surgery. The rate of body weight decrease over the first month after surgery was significantly greater in the BOFJ and Non-BOFJ group

**Fig. 4:**
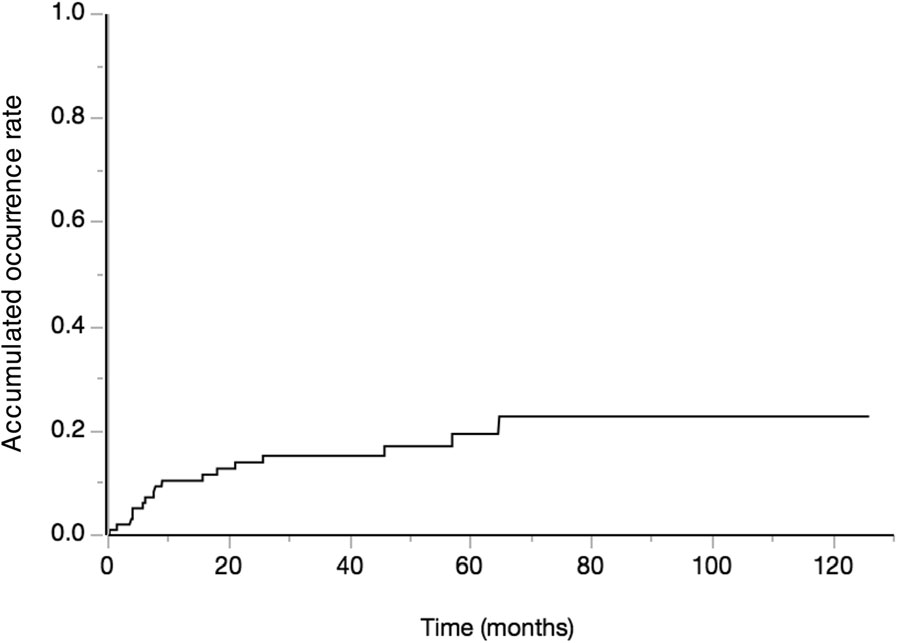
Kaplan-Meier curve showing accumulated occurrence rate of BOFJ and time (months) after the esophagectomy

Between-group comparison is reported in Table 2. Compared to the Non-BOFJ group, the BOFJ group had a higher preoperative BW (59.8 kg versus 55.6 kg, *P* = 0.053) and BMI (23.3 kg/m^2^ versus 20.9 kg/m^2^, *P* = 0.022). A laparoscopic procedure was performed in all patients in the BOFJ group, and in 84.3% of patients in the Non-BOFJ group, although this difference between the two groups was not significant (*P* = 0.080). The total operative time and volume of blood loss, and the incidence of postoperative complications, the length of postoperative hospital stay, residual cancer, adjuvant therapy, and cancer recurrence were not different between the two groups. The delay between esophagectomy and removal of the feeding catheter was also not different between the two groups (43 days versus 67 days for the Non-BOFJ and BOFJ group, respectively; *P* = 0.636). Postoperative BW (kg) was not different between the two groups, but the rate of BW decrease, from the preoperative BW, was greater in the BOFJ than Non-BOFJ group over the first month after surgery (Fig. 3). Fig. 4 shows the accumulated occurrence rate with Kaplan-Meier estimates. The cut-off value of preoperative BMI to predict the occurrence of bowel obstruction was evaluated as 23.8 with receiver operating characteristic curve.

**Table 2:** Comparison of the outcomes between the two groups

	BOFJ (*n* = 17)	Non-BOFJ (*n* = 83)	*P* value
Sex, Male, n (%)	14 (82.4)	67 (80.7)	1.000
Age, years, median (range)	67 (52–85)	67 (43–81)	0.639
Stage I / II / III / IV, n	5 / 6 / 5 / 1	20 / 19 / 32 / 12	0.823
Neoadjuvant chemotherapy, n (%)	10 (58.8)	64 (77.1)	0.117
Preoperative body weight, median (range), (kg)	59.8 (43.1–75.9)	55.6 (39.9–78.0)	0.053
Preoperative BMI, median (range), (kg / m^2^)	23.3 (19.3–29.3)	20.9 (15.1–30.0)	0.022
Laparoscopic procedure, n (%)	17 (100.0)	70 (84.3)	0.080
Operative time, median (range), (min)	591 (456–825)	613 (473–859)	0.891
Blood loss, median (range), (mL)	170 (50–490)	170 (40–1600)	0.920
Complications, n (%)			
Pneumonia	1 (5.9)	11 (13.3)	0.394
Anastomotic leakage	1 (5.9)	11 (13.3)	0.394
Recurrent nerve palsy	4 (23.5)	24 (28.9)	0.652
Surgical site infection	3 (17.7)	18 (21.7)	0.710
Hospital stay, median (range), (days)	17 (13–47)	19 (10–201)	0.505
Residual cancer, n (%)	3 (17.7)	8 (9.6)	0.392
Adjuvant therapy, n (%)	11 (64.7)	43 (51.8)	0.426
Cancer recurrence, n (%)	2 (11.8)	24 (28.9)	0.142
Postoperative weight, median (range), (kg)			
1 months	55.2 (38.0–71.0)	52.0 (38.0–74.0)	0.317
3 months	51.5 (34.0–68.0)	50.0 (33.5–73.0)	0.418
6 months	50.0 (36.0–64.0)	48.3 (35.4–70.0)	0.605
12 months	49.0 (37.0–63.0)	52.0 (36.0–70.0)	0.837
Postoperative weight rate, median (range), (%)			
1 months	90.1 (84.4–97.5)	93.8 (80.8–109.2)	0.018
3 months	84.2 (76.9–91.6)	89.3 (74.4–102.6)	0.002
6 months	82.5 (73.7–88.7)	87.0 (71.1–105.7)	0.001
12 months	80.4 (69.3–93.6)	88.9 (64.0–111.8)	< 0.001
Patient’s stature			
Length of the abdominal axis, median (range), (mm)	325 (265–380)	330 (270–380)	0.624
Distance between the site of jejunostomy and midline, median (range), (mm)	40 (22–63)	48 (20–70)	0.011
Distance between the site of jejunostomy and navel line, median (range), (mm)	20 (5–75)	35 (0–150)	0.240
Distance between the site of jejunostomy and xiphoid process line, median (range), (mm)	100 (60–180)	110 (50–180)	0.051

*BOFJ*; bowel obstruction associated with a feeding jejunostomy, BMI; body mass index

In the patient’s stature, the BOFJ patients had significantly shorter distance between the site of jejunostomy and midline (40 mm versus 48 mm, *P* = 0.011), and shorter distance between the site of jejunostomy and xiphoid process line (100 mm versus 110 mm, *P* = 0.051), compared to those in the non-BOFJ group. On multivariate analysis, higher preoperative BMI (odds ratio (OR) = 9.248; 95% confidence interval (CI) = 1.344–63.609; *p* = 0.024), higher postoperative weight loss at 3 months after the esophagectomy (OR = 8.490; 95% CI = 1.765–40.837, *p* = 0.008), and shorter distance between the site of jejunostomy and midline (OR = 8.160; 95% CI = 1.675–39.747, *p* = 0.009) were independently associated with BOFJ (Table 3).

**Table 3:** Multivariate analysis of patients with BOFJ

	Odds ratio	95% Confidence interval	*P* value
Preoperative body weight > 59.8 kg	2.062	0.352–12.089	0.422
Preoperative BMI > 23.8 kg / m^2^	9.248	1.344–63.609	0.024
Postoperative BW loss at 1 months > 10%	1.279	0.300–5.446	0.740
Postoperative BW loss at 3 months > 15%	8.490	1.765–40.837	0.008
Distance between the site of jejunostomy and midline < 45 mm	8.160	1.675–39.747	0.009
Distance between the site of jejunostomy and xiphoid process line < 100 mm	3.862	0.930–16.043	0.063

*BOFJ* bowel obstruction associated with a feeding jejunostomy, *BMI* body mass index, *BW* body weight

## Discussion

The incidence rate of BOFJ after thoracoscopic esophagectomy was 17% in our study cohort. Patients who developed BOFJ had as significantly higher preoperative BMI and higher rate of laparoscopic procedure that patients in the Non-BOFJ group. Of note, the rate of postoperative body weight loss was greater in the BOFJ than the Non-BOFJ group. In addition, our study demonstrated that shorter distance between the jejunostomy and midline or xiphoid process line might be a risk of BOFJ.

Previous studies have reported on the importance of a feeding jejunostomy after esophagectomy to provide sufficient caloric intake to compensate for anastomotic leakage and postoperative weight loss due to insufficient oral intake after surgery [11, 12]. Although improvement in surgical technique has improved the rate of anastomotic leakage, the incidence of leakage is persisting. As such, including a feeding jejunostomy after esophagectomy provides a solution to ensure adequate caloric intake, via enteral feeding, to avoid rapid weight loss, and can be to supplement oral intake, as needed, after discharge [11]. However, jejunostomy-related complications, including BOFJ, require close monitoring and emergent treatment [13].

Laparoscopy has improved the outcomes of esophagectomy, compared to an abdominal approach, reducing the incidence of abdominal adhesions and postoperative abdominal pain, compared to laparotomy [14]. However, studies have reported that lower adhesion formation after laparoscopy might be a risk factor for postoperative BOFJ and internal hernia [15, 16]. We also need to consider that gastric mobilization creates a large intra-abdominal space, on the left side of the jejunostomy, into which the jejunum might invaginate and twist around the feeding jejunostomy. This might explain the higher rate of BOFJ among patients who underwent a laparoscopic approach, and shorter distance between the jejunostomy and midline or xiphoid process line in our study group.

In the previous report, laparoscopic procedure and fixation of the jejunum only at the catheter insertion point resulted in 11.5% of BOFJ [17]. On the other hand, open abdominal surgery and longitudinal fixation of the catheter resulted in less than 6.0% of BOFJ [18–20]. Judging from these, the reason of our high incidence of BOFJ might be fewer abdominal adhesion condition with laparoscopy and small area fixation suture around the catheter via a small abdominal incision, resulted in shorter distance between the catheter and midline, creating a large internal hernia space. Although a few reports described the risk factors of BOFJ, Choi AH reported that prolonged duration of tube feeding or internal hernia space created after the surgery might be risk-factors of BOFJ [19]. The BOFJ was caused by separation of the fixation from the jejunum and abdominal wall. After experience of BOFJ, we added some longitudinal sutures using non-absorbable silk thread at the anal side of catheter to avoid torsion of jejunum. However, Akiyama et al. reported 9.1% of BOFJ although use of a non-absorbable thread for fixation [21].

In our study cohort, patients in the BOFJ group had a higher preoperative BMI and postoperative rate of BW decrease after surgery, than the BOFJ group. The higher preoperative BMI is likely indicative of fewer symptoms of esophageal cancer, including dysphagia and pain during swallowing, and, thus, patients with a higher preoperative BMI are likely to have maintained a better oral caloric prior to surgery and, thus, to have insufficient oral intake after esophagectomy [4]. Postoperative BW loss after surgery might further be accentuated in these patients by the creation of intra-abdominal spaces, due to abdominal muscle atrophy and loss of adipose tissue. By contrast, patients with preoperative symptoms of esophageal cancer would have a lower preoperative BMI; postoperatively, however, improvements in symptoms would improve caloric intake after esophagectomy. While we consider that postoperative BW loss is an outcome of BOFJ. We found that the BW loss of BOFJ patients was higher than those of non-BOFJ patients during 3 months after the esophagectomy, and the surgery for BOFJ was performed 8.4 months (median) after the esophagectomy. We considered that the pre-BOFJ condition including adhesion or torsion of jejunum might be a cause of higher weight loss.

Despite the benefits of a feeding jejunostomy after esophagectomy, an alternative enteral feeding method would be desirable to avoid BOFJ. Some researchers have recommended insertion of the feeding catheter into the gastric tube [20, 22] or duodenum [23], through the round ligament of liver, rather than through the jejunum. However, insertion of a feeding catheter into the gastric tube requires a retro-sternum reconstruction. As such, a duodenostomy might be a better option, via a posterior-mediastinum route, because of the shorter distance from the abdominal wall, although this approach does require performance of a Kocher mobilization. It has been proposed that use of a nasoduodenum tube might provide a safe and useful alternative, avoiding the burdens of enteral feeding [24]. Since 2018, we changed the feeding catheter method from jejunostomy to duodenostomy through the round ligament.

The limitation of our study need to be acknowledged. This was a retrospective observational study, with a small sample size. We cannot deny a restricted oral intake prior to the diagnosis of BOFJ due to upper abdominal pain or epigastralgia, which would have contributed to the greater rate of body weight loss after esophagectomy. Additionally, we did not monitor caloric intake after discharge. Therefore, large-scale prospective studies are warranted to determine if a feeding jejunostomy is beneficial to maintain body weight after esophagectomy, or if it is harmful, with BOFJ restricting oral intake after esophagectomy.

## Conclusion

We identified a higher risk for BOFJ among patients with a higher preoperative BMI and shorter distance between the site of jejunostomy and midline. In addition, these patients did experience a greater rate of body weight loss over the 3 month after surgery, compared to patients who did not develop BOFJ. This is an important finding when considering that severe weight loss after esophagectomy is a known risk factor of a poor prognosis. Considering the effect of BOFJ on postoperative weight loss, there is a need to consider alternative methods of enteral feeding, including use of a duodenum tube through the round ligament or a nasoduodenum tube.

## Data Availability

The datasets during and/or analyzed during the current study available from the corresponding author on reasonable request.

## Abbreviations

BMI: Body mass index
BOFJ: Bowel obstruction associated with a feeding jejunostomy
BW: Body weight
CI: Confidence interva
CT: Computed tomography
OR: Odds ratio
TSE: Thoracoscopic esophagectomy
